# Simultaneous detection of genetic and copy number alterations in *BRCA1/2* genes

**DOI:** 10.18632/oncotarget.22962

**Published:** 2017-12-06

**Authors:** Yosuke Hirotsu, Yoshihiko Ooka, Ikuko Sakamoto, Hiroshi Nakagomi, Masao Omata

**Affiliations:** ^1^ Genome Analysis Center, Yamanashi Central Hospital, Kofu, Yamanashi 400-8506, Japan; ^2^ Department of Gastroenterology and Nephrology, Graduate School of Medicine, Chiba University, Chiba 260-8677, Japan; ^3^ Department of Obstetrics and Gynecology, Yamanashi Central Hospital, Fujimi Kofu-City, Yamanashi 400-8506, Japan; ^4^ Department of Breast Surgery, Yamanashi Central Hospital, Yamanashi 400-8506, Japan; ^5^ University of Tokyo, Hongo, Bunkyo-Ku, Tokyo 113-8655, Japan

**Keywords:** BRCA1, BRCA2, mutation, copy number, diagnosis

## Abstract

Germline mutations in *BRCA1* and *BRCA2* genes (*BRCA1/2*) predispose to hereditary breast and ovarian cancer syndrome (HBOC), and their dysregulation increases the risk of cancers. The detection of pathogenic *BRCA1/2* variants is essential for the diagnosis and prevention of HBOC, and for offering treatment decisions for patients. Therefore, there is a growing demand for the development of accurate, rapid assay systems that simultaneously detect pathogenic variants and copy number alterations. Here, we tested Thermo Fisher Scientific’s newly developed Oncomine^®^ *BRCA1/2* Panel. We showed that all mutations in standard reference DNA were detected with high accuracy, and that values of allelic fractions were detected with high concordance (*R*^2^ = 0.9986). The Oncomine^®^ *BRCA1/2* Panel detected 21 pathogenic germline variants in 147 patients with breast and/or ovarian cancer, of which 20 were detected by the previously-launched Ion AmpliSeq™ *BRCA1/2* Panel, except for one frameshift mutation. The Oncomine^®^ *BRCA1/2* Panel precisely captured one additional frameshift mutation, which is difficult to detect because of the homopolymer site. Large genomic deletion was identified in one sample, which was previously detected by multiplex ligation-dependent probe amplification. Oncomine^®^ *BRCA1/2* Panel could accurately detect pathogenic variant and copy number alteration, and be an alternative assay to investigate *BRCA1/2* germline and somatic mutations.

## INTRODUCTION

*BRCA1* and *BRCA2* (*BRCA1/2*) are tumor suppressor genes with roles in the DNA repair process through their function in homologous recombination in response to DNA double-stranded breaks [1–5]. Germline heterozygous mutations in either *BRCA1* or *BRCA2* were previously shown to increase the overall risk of breast and of ovarian cancer by 40%–65% and 15%–45%, respectively [6, 7]; therefore, genetic testing of *BRCA1/2* is used to diagnose hereditary breast and ovarian cancer syndrome (HBOC) [4, 7–9].

For cancer preventive options, risk-reducing mastectomies and salpingo-oophorectomies are effective in carriers of pathogenic *BRCA1/2* mutations [10]. The loss of function of *BRCA1/2* sensitizes cells to poly (ADP-ribose) polymerase (PARP) inhibitors [2, 11, 12], and findings from clinical trials have resulted in the PARP inhibitor olaparib being approved for maintenance treatment of women with *BRCA1*- or *BRCA2*-mutated high-grade serous ovarian cancer, fallopian tube, or primary peritoneal cancer [11, 12]. Therefore, the genetic analysis of *BRCA1/2* is important for prevention, therapeutic decision-making, and proper management of breast and ovarian cancer patients.

Diagnostic testing for pathogenic *BRCA1/2* mutations is often performed by Sanger sequencing to identify single nucleotide alterations, insertions, and deletions, while large genomic alterations may be detected by the multiplex ligation-dependent probe amplification (MLPA) assay. Sanger sequencing and MLPA are considered gold standard methods to determine the *BRCA1/2* mutation status. *BRCA1/2* mutations are scattered throughout the entire coding region, and BRCA1/2 proteins are relatively large (BRCA1: 1863, and BRCA2: 3418 amino acids) [1]. Because *BRCA1* and *BRCA2* gene contains 24 and 27 exons respectively, primer pairs or probes need to be designed at each site for Sanger sequencing and MLPA assay. When DNA samples were analyzed from many patients, it is necessary to analyze higher number of PCR amplicons. Therefore, these procedures are laborious and time-consuming.

Next-generation sequencing enables large numbers of nucleotide sequences of multiple samples to be simultaneously determined [13]. The enrichment of targeted regions is mainly conducted by PCR amplification or DNA capture hybridization, and targeted sequencing of genomic regions of interest can then be used for diagnosis in a clinical setting. With the advent of this technology, the genetic bases of cancers, Mendelian diseases, and congenital diseases have been revealed [14].

We previously analyzed the entire coding regions of *BRCA1/2* in patients with breast and ovarian cancer using the Ion AmpliSeq™ *BRCA1/2* Panel, which is based on a PCR amplification method [15, 16]. Recently, Thermo Fisher Scientific has launched a new panel: the Oncomine^®^ *BRCA1/2* Panel. In this study, we examined the performance of this panel using standard reference DNA with known *BRCA1/2* mutations and variant allelic fractions to assess the accuracy of germline and somatic mutation detection. Additionally, we used the Oncomine^®^ *BRCA1/2* Panel to detect germline mutations and copy number alterations in randomly-selected patients with breast and/or ovarian cancer, and compared our findings with the sequencing results of the Ion AmpliSeq™ *BRCA1/2* Panel.

## RESULTS

### Accuracy of *BRCA1/2* mutation detection

**Figure 1:**
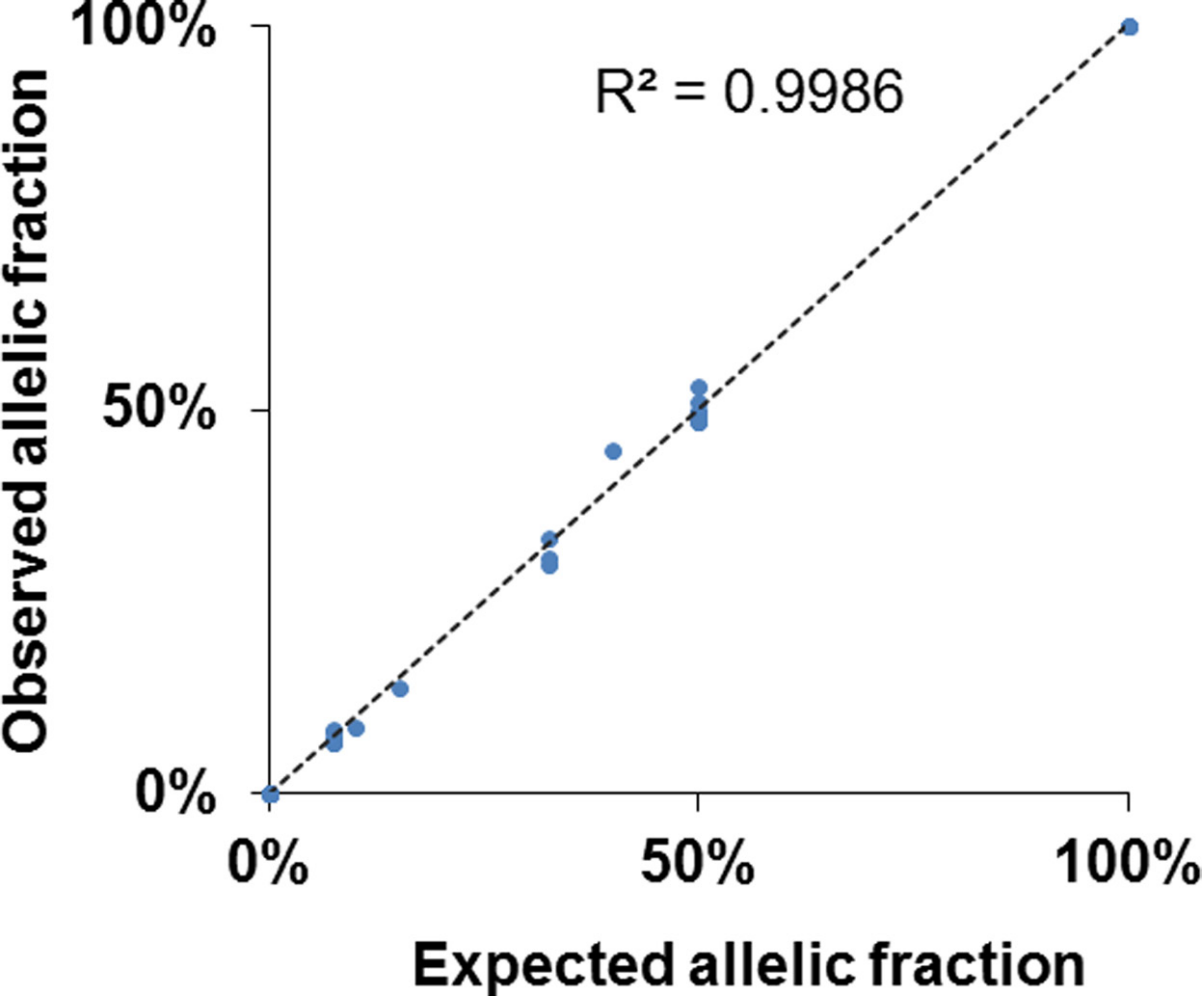
High accuracy detection of *BRCA1/2* mutations using the Oncomine^®^ *BRCA1/2* Panel The dot plot shows the variant allelic fraction between expected values in standard samples (Horizon) and observed values using the Oncomine^®^ *BRCA1/2* Panel. Mutations were detected with a high level of accuracy and concordance (decision coefficient, *R*^2^ = 0.9986).

To assess the sensitivity and specificity of mutation detection using the Oncomine^®^ *BRCA1/2* Panel (Supplementary Table 1), we first used three standard reference DNAs (HD793, HC794, and HD795) purchased from Horizon (Cambridge, UK). These DNAs harbor either wild-type or mutated *BRCA1/2* at a pre-verified allelic fraction of each mutated allele. We performed next-generation sequencing and obtained an average coverage depth of 2315× (range, 2103–2557×) (Supplementary Table 2). All mutations in the standard reference DNA were identified using the Oncomine^®^ *BRCA1/2* Panel (Table 1). Additionally, the observed variant allelic fraction significantly correlated with high accuracy (*R*^2^ = 0.9986) (Figure 1). These results show that the Oncomine^®^ *BRCA1/2* Panel is a highly accurate method of analyzing mutations with wide-ranging allelic fractions.

**Table 1:** BRCA1/2 variant detection in standard reference DNA using the Oncomine^®^ BRCA panel

Gene	Mutation	Expected Allelic Frequency (HD795)	Observed Allelic Frequency	Position	Reference	Genotype	Coding
*BRCA1*	p.S1613G	7.5%	6.7%	*chr17:41223094*	*T*	*T/C*	c.4900A>G
*BRCA1*	p.K1183R	7.5%	7.4%	*chr17:41244000*	*T*	*T/C*	c.3548A>G
*BRCA1*	p.K820E	7.5%	8.5%	*chr17:41245090*	*T*	*T/C*	c.2458A>G
*BRCA1*	p.R1443X	32.5%	30.8%	chr17:41234451	G	G/A	c.4327C>T
*BRCA1*	p.D435Y	7.5%	7%	*chr17:41246245*	*C*	*C/A*	c.1303G>T
*BRCA1*	p.P871L	15%	14%	*chr17:41244936*	*G*	*G/A*	c.2612C>T
*BRCA2*	p.N289H	7.5%	8%	*chr13:32906480*	*A*	*A/C*	c.865A>C
*BRCA2*	p.V2466A	100%	100%	*chr13:32929387*	*T*	*C/C*	c.7397T>C
*BRCA2*	p.N991D	7.5%	6.6%	chr13:32911463	A	A/G	c.2971A>G
*BRCA2*	p.K1691fs	32.5%	33.4%	*chr13:32913558*	*CA*	*CA/C*	c.5067_5067delA
*BRCA2*	p.N1784fs	40%	44.7%	*chr13:32913836*	*CA*	*CA/C*	c.5345_5345delA
*BRCA2*	p.D1420Y	32.5%	29.9%	*chr13:32912750*	*G*	*G/T*	c.4258G>T
*BRCA2*	p.I2675fs	10%	8.8%	*chr13:32937354*	*T*	*T/TA*	c.8015_8016insA

Gene	Mutation	Expected Allelic Frequency (HD793)	Observed Allelic Frequency	Position	Reference	Genotype	Coding
*BRCA1*	p.S1613G	50%	49.5%	*chr17:41223094*	*T*	*T/C*	c.4900A>G
*BRCA1*	p.K1183R	50%	50%	*chr17:41244000*	*T*	*T/C*	c.3548A>G
*BRCA1*	p.K820E	50%	49.7%	*chr17:41245090*	*T*	*T/C*	c.2458A>G
*BRCA1*	p.R1443X	0%	0%	*-*	*-*	*-*	-
*BRCA1*	p.D435Y	50%	48.7%	*chr17:41246245*	*C*	*C/A*	c.1303G>T
*BRCA1*	p.P871L	100%	100.0%	*chr17:41244936*	*G*	*A/A*	c.2612C>T
*BRCA2*	p.N289H	50%	51%	*chr13:32906480*	*A*	*A/C*	c.865A>C
*BRCA2*	p.V2466A	100%	100%	*chr13:32929387*	*T*	*C/C*	c.7397T>C
*BRCA2*	p.N991D	50%	48.4%	*chr13:32911463*	*A*	*A/G*	c.2971A>G
*BRCA2*	p.K1691fs	0%	0%	*-*	*-*	*-*	-
*BRCA2*	p.N1784fs	50%	53.1%	*chr13:32913836*	*CA*	*CA/C*	c.5345_5345delA
*BRCA2*	p.D1420Y	0%	0%	*-*	*-*	*-*	-
*BRCA2*	p.I2675fs	0%	0%	*-*	*-*	*-*	-

Gene	Mutation	Expected Allelic Frequency (HD794)	Observed Allelic Frequency	Position	Reference	Genotype	Coding
*BRCA1*	p.S1613G	0%	0%	*-*	*-*	*-*	-
*BRCA1*	p.K1183R	0%	0%	-	*-*	*-*	-
*BRCA1*	p.K820E	0%	0%	*-*	*-*	*-*	-
*BRCA1*	p.R1443X	0%	0%	*-*	*-*	*-*	-
*BRCA1*	p.D435Y	0%	0%	*-*	*-*	*-*	-
*BRCA1*	p.P871L	0%	0%	*-*	*-*	*-*	-
*BRCA2*	p.N289H	0%	0%	-	*-*	*-*	-
*BRCA2*	p.V2466A	100%	100.0%	*chr13:32929387*	*T*	*C/C*	c.7397T>C
*BRCA2*	p.N991D	0%	0%	*-*	*-*	*-*	-
*BRCA2*	p.K1691fs	0%	0%	*-*	*-*	*-*	-
*BRCA2*	p.N1784fs	0%	0%	*-*	*-*	*-*	-
*BRCA2*	p.D1420Y	0%	0%	*-*	*-*	*-*	-
*BRCA2*	p.I2675fs	50%	48.5%	*chr13:32937354*	*T*	*T/TA*	c.8015_8016insA

### Comparison of the panel designs and performance

**Figure 2:**
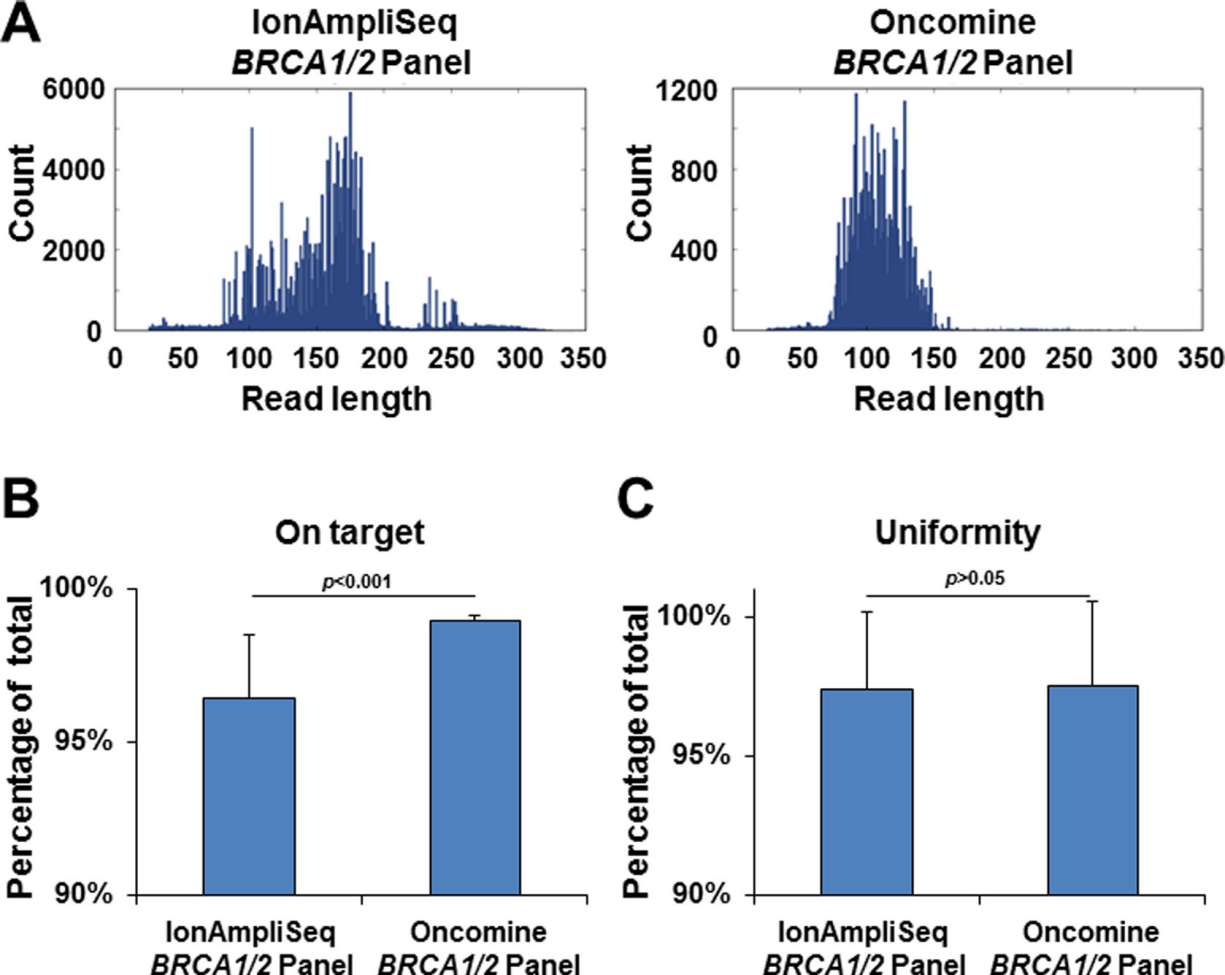
Next-generation sequencing read length and quality control (**A**) Histogram of sequencing read lengths using the IonAmpliSeq™ *BRCA1/2* Panel (left) and Oncomine^®^ *BRCA1/2* Panel (right). This is a representative image from the sequencing data. (**B**) Percentage of mapped reads that were aligned over the target region. *P*-value was calculated by the Student’s *t*-test. (**C**) Percentage of target base coverage at least >0.2× the mean coverage depth. *P*-value was calculated by the Student’s *t*-test.

We tested 147 buffy coat samples from patients with breast and/or ovarian cancer to detect germline variants by next-generation sequencing using the IonAmpliSeq™ *BRCA1/2* Panel or Oncomine^®^ *BRCA1/2* Panel. The average coverage depths obtained were 555× (range, 107–7865×) and 840× (range, 159–2484×), respectively (Supplementary Table 3). The sequencing read length was relatively short in the Oncomine^®^ *BRCA1/2* Panel (range, 65–137 bp) compared with the IonAmpliSeq™ *BRCA1/2* Panel (range, 71–239 bp) (Figure 2A). Overall, sequencing read mapping on the targeted region (mean ± SD: 98.9% ± 0.2% vs 96.5% ± 2.1%, *p* < 0.001) was significantly higher with the Oncomine^®^ *BRCA1/2* Panel than the IonAmpliSeq™ *BRCA1/2* Panel (Figure 2B). Uniformity (97.5% ± 3.0% vs 97.4% ± 2.8%, *p* < 0.05) was comparable between two panels (Figure 2C).

### Comparison of the *BRCA1/2* panels

**Figure 3:**
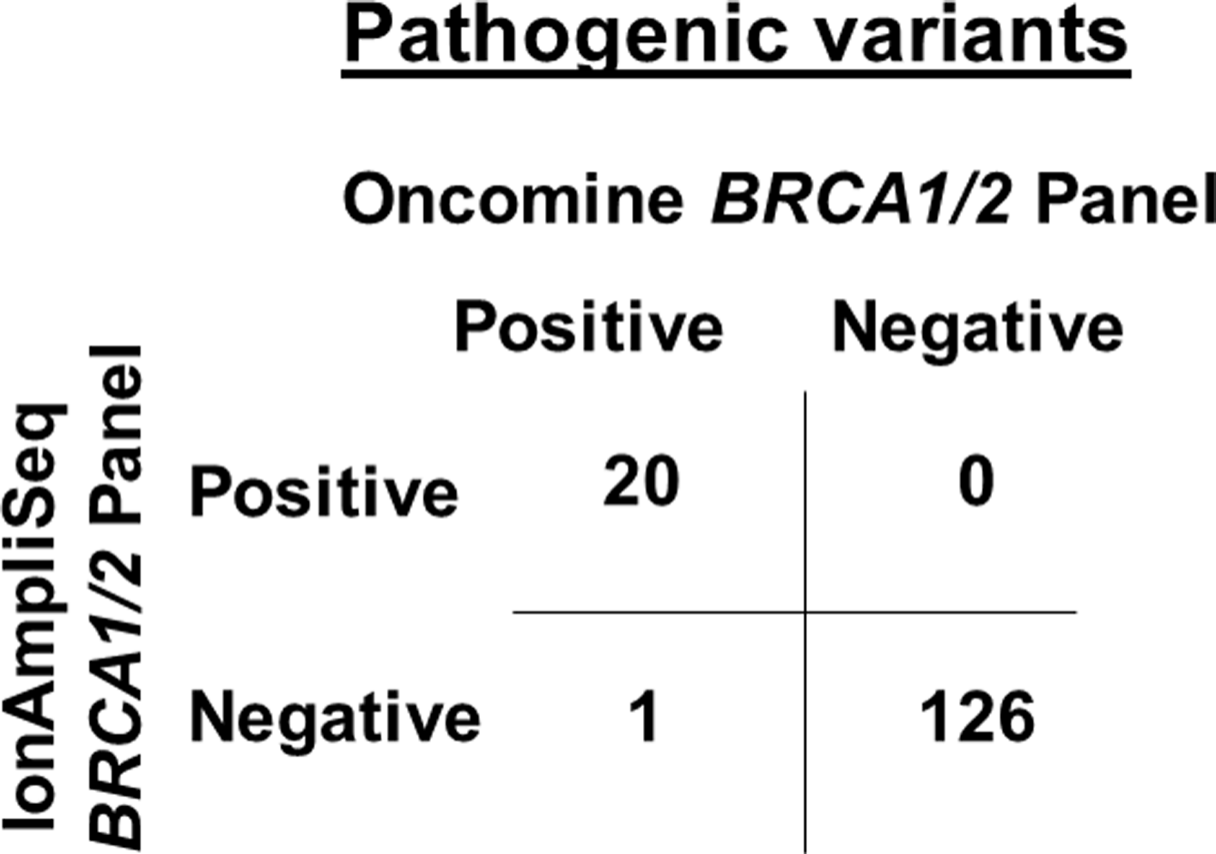
Comparison of pathogenic *BRCA1/2* variants calling between the IonAmpliSeq™ *BRCA1/2* Panel and Oncomine^®^ *BRCA1/2* Panel from 147 patients with breast and/or ovarian cancer An overall agreement of 99.3% was achieved between the two panels, with 100% sensitivity and 95.2% specificity.

The Ion AmpliSeq™ *BRCA1/2* Panel detected 20 pathogenic *BRCA1/2* variants in 147 patients (five mutations in *BRCA1*; 15 mutations in *BRCA2*) (Table 2). By contrast, the Oncomine^®^ *BRCA1/2* Panel detected 21 pathogenic variants (five variants in *BRCA1*; 16 variants in *BRCA2*) (Table 2). There was 99.3% agreement between the IonAmpliSeq™ *BRCA1/2* Panel and Oncomine^®^ *BRCA1/2* Panel, with 100% sensitivity and 95.2% specificity (Figure 3 and Table 2).

**Table 2:** Pathogenic variants detected by IonAmpliSeq™ *BRCA1/2* panel and Oncomine^®^ *BRCA1/2* panel

					IonAmpliSeq *BRCA1/2* Panel				Oncomine *BRCA1/2* Panel			
Case #	Gene	Mutation	Reference	Variant	Variant reads	Reference reads	Coverage	Allelic fraction	Variant reads	Reference reads	Coverage	Allelic fraction
Case 1	*BRCA1*	p.L63X	*A*	*T*	291	273	564	52%	155	179	334	46%
Case 2	*BRCA1*	p.K652fs	*T*	*TC*	203	245	448	45%	175	242	417	42%
Case 3	*BRCA1*	p.Q934X	*G*	*A*	211	189	400	53%	62	76	138	45%
Case 4	*BRCA1*	p.Q934X	*G*	*A*	242	241	483	50%	78	77	155	50%
Case 5	*BRCA1*	p.E1257fs	*CCT*	*C*	215	231	446	48%	92	83	175	53%
Case 6	*BRCA2*	p.Q609X	*C*	*T*	665	718	1383	48%	352	550	902	39%
Case 7	*BRCA2*	p.E790fs	*AG*	*A*	584	573	1157	50%	594	647	1241	48%
Case 8	*BRCA2*	p.Q850fs	*A*	*ACC*	78	81	159	49%	148	217	365	41%
Case 9	*BRCA2*	p.Q864fs	*AT*	*A*	185	212	397	47%	83	73	156	53%
Case 10	*BRCA2*	p.S1882X	*C*	*A*	87	77	164	53%	186	194	380	49%
Case 11	*BRCA2*	p.N1287fs	*GA*	*G*	213	291	504	42%	123	94	217	57%
Case 12	*BRCA2*	p.T1388fs	*TTTAAC*	*T*	811	873	1684	48%	99	142	241	41%
Case 13	*BRCA2*	p.S1882X	*C*	*A*	137	132	269	51%	182	197	379	48%
Case 14	*BRCA2*	p.S1882X	*C*	*A*	446	486	932	48%	132	133	265	50%
Case 15	*BRCA2*	p.N2135fs	*ATAACT*	*A*	123	173	296	42%	67	76	143	47%
Case 16	*BRCA2*	p.I2149fs	*CTA*	*C*	618	654	1272	49%	88	103	191	46%
Case 17	*BRCA2*	p.I2149fs	*CTA*	*C*	392	359	751	52%	109	129	238	46%
Case 18	*BRCA2*	p.G2281fs	*TAGAG*	*T*	210	234	444	47%	56	64	120	47%
Case 19	*BRCA2*	p.R2318X	*C*	*T*	29	62	91	32%	38	52	90	42%
Case 20	*BRCA2*	p.I2675V	*A*	*G*	97	76	173	56%	76	88	164	46%
Case 21	*BRCA2*	p.K3032fs	*CAAAA*	*C*	ND	ND	ND	ND	204	196	400	51%

ND, not detected

### Validation of discordant results

**Figure 4:**
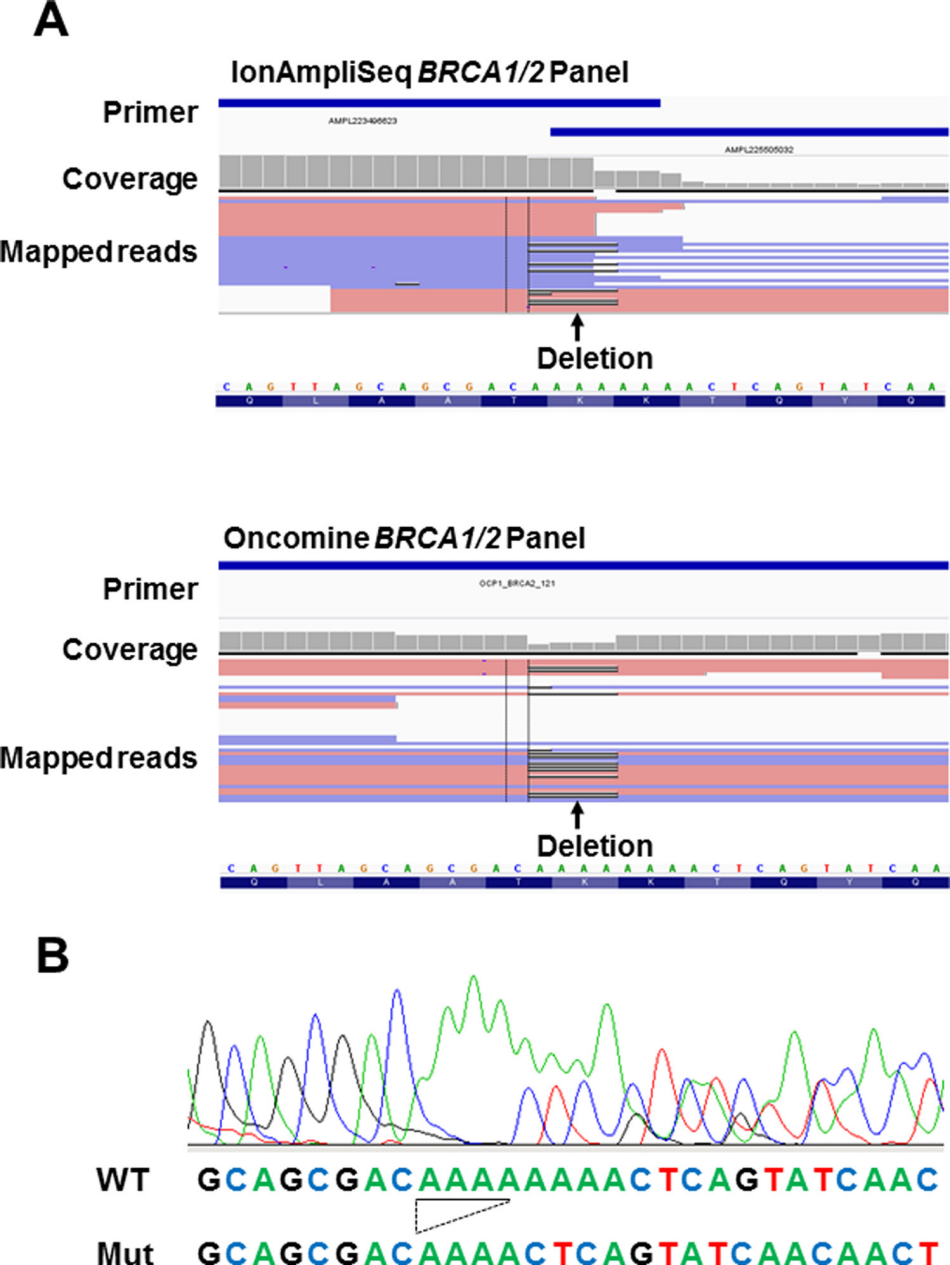
Analysis of the discordant sample (**A**) Read alignment of the discrepant result between the IonAmpliSeq™ *BRCA1/2* Panel (top) and Oncomine^®^ *BRCA1/2* Panel (bottom) in case 21. Arrow indicates the position of the deletion (*BRCA2* p.K3032fs; c. 9090_9093delAAAA, black line in mapped reads data). The primer position is indicated by a blue line at the top of the image. The mutation missed by the IonAmpliSeq™ *BRCA1/2* Panel is located at the end of the primer position. (**B**) Diagram of Sanger sequencing data. The *BRCA2* p.K3032fs mutation was detected in case 21. This mutation was detected using next-generation sequencing data of the Oncomine^®^ *BRCA1/2* Panel, but not using the IonAmpliSeq™ *BRCA1/2* Panel with default variant call algorithm parameters. The nucleotide sequences of wild-type (WT) and mutant (Mut) are given at the bottom. The deletion is underlined.

Among the 147 patients, pathogenic *BRCA1/2* variants were observed in 20 by both the Oncomine^®^ *BRCA1/2* Panel and IonAmpliSeq™ *BRCA1/2* Panel. However, the novel pathogenic frameshift variants *BRCA2* p.K3032fs was only detected by the Oncomine^®^ *BRCA1/2* Panel in one patient. The mapped sequencing read visualized by the IGV revealed nucleotide deletion reads (Figure 4A). To validate this discordant result between the two panels, we performed Sanger sequencing and detected *BRCA2* p.K3032fs in the patient (Figure 4B).

We next examined the reason for the false-negative result from the IonAmpliSeq™ *BRCA1/2* Panel. The *BRCA2* p.K3032fs mutation is a 4-bp deletion (c. 9090_9093delAAAA) at the eight poly (A) homopolymer site (Figure 4B), which is located near the end of the amplicon (Figure 4A). Therefore, the sequencing reads may have been filtered out during the mapping reads process because of a low read quality or because they were outside the targeted region. The IonAmpliSeq™ *BRCA1/2* Panel did not detect the mutation using default variant call algorithm parameters, so we optimized this to enable *BRCA2* p.K3032fs detection. However, a low allelic fraction and low-depth data remained (allelic fraction: 38%, variant read/total read = 28/73). This suggests that a low-depth region and low-quality base score increases the risk of obtaining a false-negative result. In contrast, the Oncomine^®^ *BRCA1/2* Panel accurately detected *BRCA2* p.K3032fs, perhaps because of the inclusion of a higher number of amplicons at the target region.

### Copy number analysis

**Figure 5:**
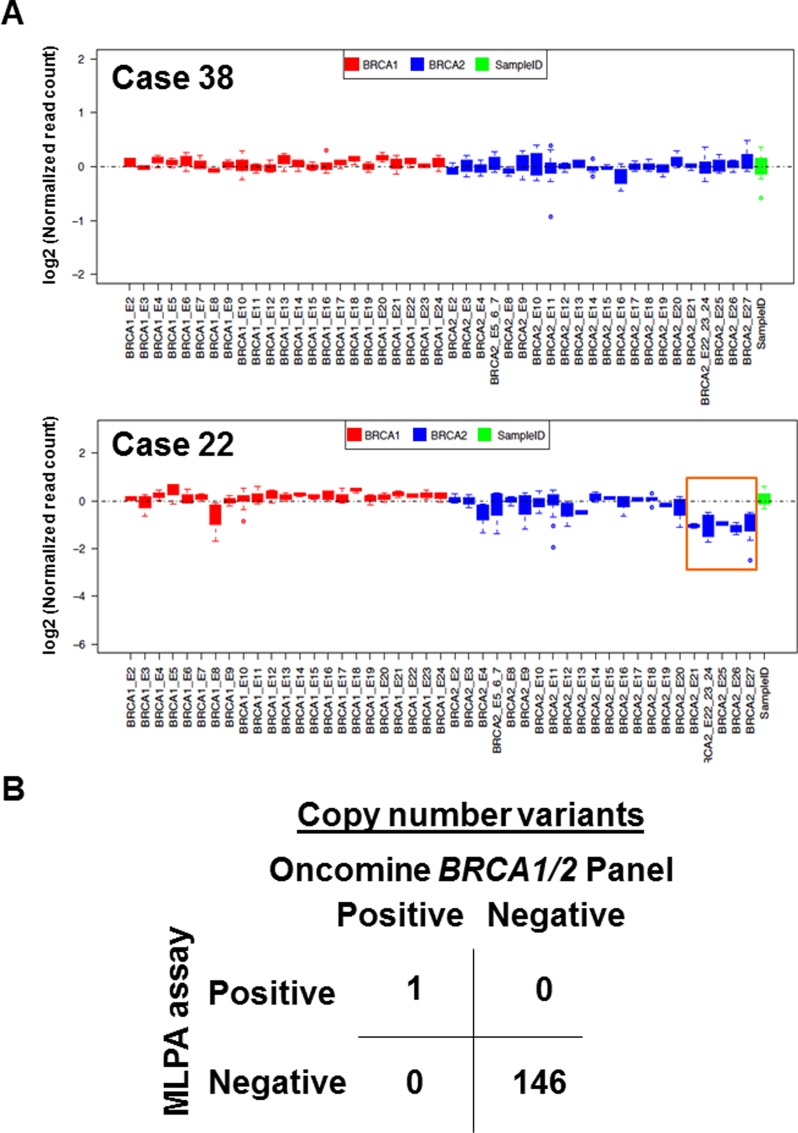
Copy number analysis identified a large genomic deletion (**A**) Representative images from a patient with no copy number alteration (upper, case 38) and with a *BRCA2* exon 21-27 deletion (lower, case 22). The orange box indicates the deletion locus. Sequencing reads of *BRCA1* (red) and *BRCA2* (blue) loci were normalized with SampleID tag sequencing reads (green). **(B**) Comparison of copy number alterations between the MLPA assay and Oncomine^®^ *BRCA1/2* Panel from 147 patients with breast and/or ovarian cancer. An overall agreement of 100% between the two assays was achieved, with 100% sensitivity and 100% specificity.

A previous study demonstrated that the IonAmpliSeq™ *BRCA1/2* Panel is not a feasible method for analyzing copy number alterations because of the high number of false-positive results obtained, and the fact that an MLPA assay is required [17]. We therefore examined whether the Oncomine^®^ *BRCA1/2* Panel is suitable for analyzing copy number alterations. In our series, a large *BRCA1/2* genomic deletion had already been determined by the MLPA assay, and a *BRCA2* exon 21 to 27 deletion had been identified in one patient (case 22) [16]. To determine the copy number variation in 147 patients, we used sequencing reads data from the Oncomine^®^ *BRCA1/2* panel and normalized SampleID sequencing reads from eight different loci. Among 147 patients, one (case 22) was shown to carry a large *BRCA2* genomic deletion at exon 21 to 27, which is consistent with MLPA assay findings (Figure 5A and Supplemental Table 4). Thus, there was 100% agreement between the MLPA assay and Oncomine^®^ *BRCA1/2* Panel (Figure 5B and Supplemental Table 4). These data demonstrate that the Oncomine^®^ *BRCA1/2* Panel accurately detects copy number variants.

## DISCUSSION

In this study, we used an early accessed version of the Oncomine^®^ *BRCA1/2* Panel and compared its performance in next-generation sequencing with that of the IonAmpliSeq™ *BRCA1/2* Panel on the Ion PGM system. To our knowledge, this is the first direct comparison of the Oncomine^®^ *BRCA1/2* Panel and IonAmpliSeq™ *BRCA1/2* Panel. Our results demonstrated Oncomine^®^ *BRCA1/2* Panel enable us to simultaneously detect the germline variant and copy number alterlations. Oncomine^®^ *BRCA1/2* Panel accurately detects pathogenic *BRCA1/2* variants in patients with breast and/or ovarian cancer, so is feasible for use in cancer genome research and diagnostic purposes in a clinical setting.

Germline mutations in Mendelian disease genes or predisposed genes for hereditary cancers have been identified in the previous studies, and the detection of these is used for the clinical diagnosis of disease. Sanger sequencing, the gold standard method, is widely used for detecting germline mutations. However, it is expensive and laborious, especially when analyzing long-range targeted regions without hotspot mutation sites [18].

BRCA1/2 encodes proteins with a high number of amino acids, while pathogenic variants are scattered throughout the coding regions. To overcome these problems, next-generation sequencing technology can be used for genomic analyses, and a range of assay panels are available for amplicon-based targeted next-generation sequencing, including IonAmpliSeq™ and the Oncomine^®^ *BRCA1/2* Panel (Thermo Fisher Scientific), BRCA MASTR Dx (Multiplicom, Niel, Belgium), TruSeq (Ilumina, San Diego, CA, USA), GeneRead Human *BRCA1/2* Panel (Qiagen), HaloPlex (Agilent Technologies, Santa Clara, CA), and custom-designed panels [18–23]. These technologies offer a powerful tool for use in clinical oncology.

In the present analysis, we investigated the sequencing performance of the Oncomine^®^ *BRCA1/2* Panel by comparing data with those obtained from the previously available IonAmpliSeq™ *BRCA1/2* Panel [15]. The Oncomine^®^ *BRCA1/2* Panel was capable of identifying a deletion at a homopolymer site, which was not detected by the IonAmpliSeq™ *BRCA1/2* Panel. The on target rate of mapping reads was significantly higher using the Oncomine^®^ *BRCA1/2* Panel than the IonAmpliSeq™ Panel (Figure 2B). Thus, the sequencing reads were mapped across the board target region. Recently, false-negative variant calling was reported in a study in which primers were designed to amplify a rare single nucleotide polymorphism, because the single nucleotide polymorphism was located in the primer-binding site [22]. This result suggests that a sufficient number of amplicons, as included in the Oncomine^®^ *BRCA1/2* Panel, decreases the possibility of obtaining low-depth regions, low-quality mapped reads, and false-negative results.

When 8 samples were analyzed in a reaction, the running cost per sample is 34,000 JPY (340 USD) for Oncomine^®^ BRCA1/2 Panel, 19,000 JPN (190 USD) for Ion AmpliSeq™ BRCA1/2 Panel and 190,000 JPY (1,900 UDS) for Sanger sequencing. Although the cost of Oncomine^®^ BRCA1/2 Panel is slightly higher than that of Ion AmpliSeq™ BRCA1/2 Panel, single nucleotide variant, small indel and CNV were simultaneously detected with streamlined workflow using Oncomine^®^ BRCA1/2 Panel and IonReporter Software. Additionally, the Oncomine^®^ *BRCA1/2* Panel includes internal control primer amplicons, so copy number analysis is feasible without the need for other reference standards. Thus, next-generation sequencing using the Oncomine^®^ *BRCA1/2* Panel will obtain genetic information about single nucleotide variants and copy number variation while reducing turnaround times and costs. This improved design of assay panel could therefore become an alternative or replacement for Sanger sequencing and the MLPA assay.

Germline or somatic mutations, copy number loss, and DNA methylation frequently occur in *BRCA1/2* in breast and ovarian cancer [24, 25]. These genetic and epigenetic alterations diminish BRCA1/2 functions and dysregulate the DNA repair pathway. PARP inhibitors were previously shown to have a synthetically lethal therapeutic effect on BRCA-deficient tumor cells [2]. PARP inhibitors have therefore been approved for use in advanced ovarian cancer patients harboring germline *BRCA1/2* mutations in both the USA and Europe, but not in Japan yet. Given that this therapy will be approved for patients with BRCA-deficient cancer caused by somatic events, diagnostic demand is expected to increase for the analysis of *BRCA1/2* somatic mutations and copy number alterations. The simultaneous detection of mutations and copy number alterations is an attractive and useful prospect for clinical settings. Therefore, the Oncomine^®^ *BRCA1/2* Panel offers an improved and accurate assay method that is likely to accelerate the sequential procedures of genetic counseling [26], diagnosis, prevention, and treatment decisions for eligible patients.

## METHODS

### Early access program

Since April 2016, our genome analysis center has participated in the early access program and used early access version of Oncomine^®^ *BRCA1/2* Panel. Program participants received free primer pools targeting *BRCA1/2* genes from Thermo Fisher Scientific (Waltham, MA).

### Sample preparation and patients

For standard genomic DNA samples, BRCA Germline I (HD793), BRCA Germline II (HD794) and BRCA Somatic Multiplex (HD795) were purchased from Horizon (Cambridge, UK). Peripheral blood samples were obtained from 147 breast and/or ovarian cancer patients who attended our hospital [15, 16]. Buffy coats were isolated following centrifugation of peripheral blood samples and freezed at −80°C until DNA extraction. Buffy coat DNA was extracted using the QIAamp DNA Blood Mini QIAcube Kit (Qiagen, Hilden, Germany) with the QIAcube (Qiagen). The concentration of DNA was determined using the Nano Drop 2000 spectrophotometer (Thermo Fisher Scientific). All patients provided written informed consent for the genetic research study, which was performed in accordance with the protocols approved by the Institutional Review Board at Yamanashi Central Hospital according to the relevant national guidelines.

### Next generation sequencing

We used The Ion AmpliSeq™ *BRCA1/2* Panel containing 167 primer pairs in three pools and the Oncomine^®^ *BRCA1/2* Panel containing 275 primer pairs in two pools (Supplementary Table 1). These two panels cover all exons of *BRCA1/2* coding regions and exon–intron boundaries. The amount of DNA required for the IonAmpliSeq™ *BRCA1/2* Panel is 30 ng, while 20 ng is needed for the Oncomine^®^ *BRCA1/2* Panel. This latter panel also uses a lower number of primer pools, therefore enabling the analysis to be performed in a less laborious manner during library preparation.

To compare with performances between these panels, previously published data of The Ion AmpliSeq™ *BRCA1/2* Panel was used [15, 16]. Targeted sequencing using Oncomine^®^ *BRCA1/2* Panel was performed according to the manufacturer’s protocol. In brief, multiplex PCR was performed with a premixed primer pool using Ion AmpliSeq Library Kit 2.0 (Thermo Fisher Scientific). Multiplex PCR condition is as follow: 99°C for 2 min; 18 cycles of 99°C for 15 sec, 60°C for 4 min; and 10°C for hold (16 hours maximum). PCR amplicons of each primer pool were combined and partially digested primer sequences with 2μl FuPa reagent as following condition: at 50°C for 10 min; 55°C for 10 min; 60°C for 20 min. Amiplicon product was ligated to barcodes adaptors with IonXpress Barcode kit (Thermo Fisher Scientific) for 30 min at 22°C then 72°C for 10 min. Adaptor ligated libraries were purified using Agencourt AMPure XP reagents (Beckman Coulter, Brea, CA). The library concentration was determined using an Ion Library Quantitation Kit (Thermo Fisher Scientific). Emulsion PCR was carried out using the Ion OneTouch™ System and Ion PGM™ Hi-Q™ OT2 Kit (Thermo Fisher Scientific) according to the manufacturer’s instructions. Next generation sequencing was performed on the Ion PGM system using Ion PGM Hi-Q Sequencing Kit (Thermo Fisher Scientific).

### Data analysis

Sequence data were processed using standard Ion Torrent Suite Software running on the Torrent Server. Raw signal data were analyzed using Torrent Suite version 5.0.4. The data processing pipeline involved signaling processing, base calling, quality score assignment, adapter trimming, PCR duplicate removal, read alignment to the human genome 19 reference (hg19), quality control of mapping quality, coverage analysis [27, 28]. Following data analysis, the annotation of single nucleotide variants, insertions, and deletions was performed by the Ion Reporter Server System (Thermo Fisher Scientific) [19, 29]. For Standard Reference Germline I and Germline II DNA (HD793 and HD794) and DNA extracted from 147 patient’ lymphocyte, germline variant calling parameter were used (Single sample analysis, Variant Type Detection = Germline default parameter). For BRCA Somatic Multiplex DNA, somatic variant calling parameter were used (Single sample analysis, Variant Type Detection = Somatic default parameter, Indel Min Variant allelic fraction >0.05). The minimum count for mutant allele reads was ≥5, the coverage depth was ≥30. Splice site alternations were analyzed 2 bp upstream or downstream of exon–intron boundaries. Pathogenic variants were defined as mutations causing protein-truncating mutations (nonsense, frameshift insertion, frameshift deletion) or splice site mutation [15]. Sequence data were visually confirmed with the Integrative Genomics Viewer (IGV) and any sequence, alignment, or variant call error artifacts were discarded. We followed the term and interpretation of sequence variants recommended in the American College of Medical Genetics and Genomics (ACMG) guidelines [30].

### Copy number analysis

Copy number variants were determined according to the original algorithm pipeline developed by Thermo Fisher Scientific. SampleID (8 amplicons amplified different chromosomes loci) was used as internal control to normalize data. To determine the sensitivity and specificity, copy number data was compared with the results of the MLPA assay (MRC-Holland, Amsterdam, the Netherlands). MLPA assay has been already performed using buffy coat DNA as previously described [16].

### Sanger sequencing

One discordant sequencing data was validated by Sanger sequencing. PCR was performed using DNA as a template and primer pairs flanking the pathogenic variant sites (*BRCA2* p.K3032fs). Forward primer: (5′-GATGTCACAACCGTGTGGAA-3′) and reverse primer (5′-GCCAACTGGTAGCTCCAACTAA-3′) were used in PCR reaction. PCR products were separated on 2% agarose gel electrophoresis to confirm PCR products and purified using the Agencourt AMPure XP reagents (Beckman coulter, Brea, CA) according to the manufacturer’s instructions. Sequencing was performed with BigDye Terminator v3.1 using the same forward or reverse primers used for first PCR amplification [31]. Sequencing PCR products were purified and subsequently analyzed by the 3500 Genetic Analyzer (Thermo Fisher Scientific). GenBank sequences of human BRCA1 (RefSeq accession number: NM_007294.3 and NP_009225.1) and BRCA2 (RefSeq accession number: NM_000059.3 and NP_000050.2) were referred to at the NCBI Reference Sequence Database.

## SUPPLEMENTARY MATERIALS TABLES






