# Supplementary material for: Simultaneous detection of genetic and copy number alterations in BRCA1/2 genes
Source: Oncotarget. 2017 Dec 6;8(70):114463–73. doi: 10.18632/oncotarget.22962 (PMC5777706; doi:10.18632/oncotarget.22962)
Supplement: Supplementary file 1 [file oncotarget-08-114463-s001.pdf]

Simultaneous detection of genetic and copy number alterations in *BRCA1/2* genes

SUPPLEMENTARY MATERIALS

Supplementary Table 1: Comparison of *BRCA 1/2* panel information

Panel name	Number of amplicon	Number of primer pools	Input DNA required	Panel coverage*
IonAmpliSeq™ *BRCA1/2* Panel	167 pairs	3 pools	30 ng	100%
Oncomine® *BRCA1/2* Panel	275 pairs	2 pools	20 ng	100%

*Designed coverage of coding regions and exon–intron boundaries of *BRCA1* and *BRCA2*

Supplementary Table 2: Coverage analysis of standard reference samples

Sample name	Mapped reads	On target	Mean depth	Uniformity
HDx_Somatic (HD795)	588805	99.26%	2557	97.20%
HDx_Germline1 (HD793)	482858	99.32%	2103	99.15%
HDx_Germline2 (HD794)	527234	99.57%	2284	97.68%

Supplementary Table 3: Coverage analysis of buffy coat samples from breast and/or ovarian cancer patients (*n* = 147). See Supplementary_Table_3

Supplementary Table 4: Comparison of copy number analysis data (*n* = 147). See Supplementary_Table_4
